# Adding Perches for Cross-Pollination Ensures the Reproduction of a Self-Incompatible Orchid

**DOI:** 10.1371/journal.pone.0053695

**Published:** 2013-01-07

**Authors:** Zhong-Jian Liu, Li-Jun Chen, Ke-Wei Liu, Li-Qiang Li, Wen-Hui Rao, Yu-Ting Zhang, Guang-Da Tang, Lai-Qiang Huang

**Affiliations:** 1 Shenzhen Key Laboratory for Orchid Conservation and Utilization, The National Orchid Conservation Center of China and The Orchid Conservation & Research Center of Shenzhen, Shenzhen, China; 2 The Center for Biotechnology and BioMedicine, Graduate School at Shenzhen, Tsinghua University, Shenzhen, China; 3 College of Forestry, South China Agricultural University, Guangzhou, China; Tel Aviv University, Israel

## Abstract

**Background:**

Outcrossing is known to carry genetic advantages in comparison with inbreeding. In many cases, flowering plants develop a self-incompatibility mechanism, along with a floral component adaptation mechanism, to avoid self-pollination and to promote outbreeding. Orchids commonly have a lip in their flower that functions as the a visiting plate for insect pollinators. Aside from the lip, however, many species (including *Coelogyne rigida*) have sheaths around the axis of inflorescence. The function of these sheaths remains unknown, and has long been a puzzle to researchers.

**Methodology/Principal Findings:**

We investigated the function of these sheaths in relation to the lip and the pollinators, as well as their role in the modes of pollination and reproduction of *Coelogyne rigida* in 30 flowering populations of orchids in the limestone area of Southeast Yunnan, China. We found that self-incompatible *C. rigida* developed specialized bird perches around the basal axis of inflorescence to attract sunbirds and to complement their behavioral tendency to change foraging locations frequently. This self-incompatibility mechanism operates separately from the floral component adaptation mechanism. This mechanism thus prevents bees from repeatedly visiting the floral lip of the same plant which, in turn, results in autogamy. In this way, instead of preventing autogamy, *C. rigida* responds to these negative effects through a highly efficient cross-pollination method that successfully transfers pollen to different plants.

**Conclusions:**

The proposed method ensures reproductive success, while offsetting the infertile self-pollination by insects, thereby reducing mating costs and addressing the lack of cross-pollination. The adaptation provides a novel and striking example of structural adaptation that promotes cross-pollination in angiosperms.

## Introduction

The transition from outcrossing to self-fertilization is one of the most common evolutionary trends in plants [1], [2]. Although selfing commonly occurs in angiosperms [3]–[6], the detrimental effects of inbreeding that follow repeated selfing [6], [7] have promoted strong natural selection in mating systems, thus ensuring successful cross-fertilization (outcrossing) [8]. Therefore, angiosperms have developed numerous mechanisms to avoid selfing and to promote outbreeding [8], among which the most prevalent is self-incompatibility [9]. Self-incompatibility effectively mitigates the harmful effects of self-mating and inbreeding depression. However, this mechanism does not prevent self-pollination, which results in ovule and pollen discounting at a high mating cost and does not directly promote crossing. Therefore, self-incompatible plants commonly evolve through floral component adaptation mechanisms to prevent self-pollination and/or to promote cross-pollination.

Flowers provide visiting plates that enable visitors to linger on them [10] for a length of time that is sufficient to collect nectar. This process favors the transfer of pollen, which, in turn, enhances the chance of mating. Orchids usually facilitate pollinator visitation by using a specialized lip that serves as a visiting plate to enable pollination by insects. In addition to such lips, some species of *Coelogyne* have many coriaceous sheaths around the basal or apical part of the inflorescence axis. For example, *Coelogyne rigida* [11], a self-incompatible orchid, has a section of its basal axis covered with and surrounded by many sheaths. The function of these sheaths has long puzzled botanists and remains unclear.

The aims of this study are as follows: (1) to determine the role of the sheaths surrounding the inflorescence basal axis; (2) to determine how these sheaths facilitate the transfer of pollen by sunbirds; (3) to quantify the level of fruit production affected by sheaths; and (4) to determine the relationship between the sheaths and the lips so that the roles of birds and insects as pollinators in the evolution of the *C. rigida* breeding system can be assessed and compared. In addition, to gain a clear understanding of the interaction between inflorescence traits and pollinators, a comparison was made of *C. rigida* to *C. fimbriata*, a related species with an erect inflorescence lacking a sheathed perch at its base and pollinated exclusively by wasps. We investigated and characterized the role of the sheaths and their relationship with the lips in 30 flowering populations of *C. rigida*. We also found that *Aethopyga gouldiae* (sunbird), *Vespula* sp. (wasp), and *Apis cerana* (honeybee) are the pollinators of *C. rigida* in the limestone area of Southeast Yunnan, China. Therefore, we hypothesize that in response to inbreeding by insect visitation through the lips, *C. rigida* evolved sheaths on its basal inflorescence axis to function as bird perches that can facilitate the attachment of its pollen to the beak of the sunbird which, in turn, ensures pollen transfer and outbreeding.

Furthermore, we discovered that unlike the floral lip utilized for insect visitation, the sheaths around the inflorescence axis of the *C. rigida* provide perches for the sunbird, *A. gouldiae*, its effective pollinator. We then demonstrated that this structure ensures the success of plant outbreeding by guiding the sunbirds to adopt specific positions when visiting flowers for pollen dispersal. The results show that *C. rigida* does not require a change in floral components to avoid self-pollination. Rather, *C. rigida* has developed an inflorescence structural adaptation mechanism to accomplish cross-pollination efficiently through birds, thereby offsetting infertile self-pollination and compensating for the actual negligible cross-pollination by insects.

## Results

### Morphology of *C. rigida*

*C. rigida* grows on humus-covered rocks in limestone mountain forests. The flowering period of this species is from March to May. Each plant clone grows up to 10 m^2^ to 15 m^2^, with flowers opening simultaneously on 100 to 300 inflorescences ( Fig. S1). The inflorescence of *C. rigida* is apical on the old pseudobulb and is pendulous, measuring 10 cm to 20 cm long, with more than 10 imbricate coriaceous sheaths surrounding the basal axis of the inflorescence, thus forming a conical-shaped segment that is 3 cm to 4 cm long. The raceme has six to 12 bisexual flowers, and the rachis is twisted horizontally (Fig. S2). The flowers in each inflorescence open from the middle to both ends, one after another, at two-day intervals. The unpollinated inflorescence lasts for approximately 20 days.

### Observation on visiting behavior of pollinators

After 210 hours of observation during three flowering seasons, the sunbird (*A. gouldiae*), wasp (*Vespula* sp.), and honeybee (*Apis cerana*) were identified to be the pollinators that successfully visit *C. rigida*. And it was observed that sunbird totally visited 467 times and its visiting rate was 0.11(±0.02) time (n = 15 days)/inflorescence (flower)/hour; wasp visited 337 times at a visiting rate of 0.08 (±0.01) time (n = 15 days)/inflorescence/hour; and honeybee visited 372 times with its visiting rate was 0.09 (±0.01) time (n = 15 days)/inflorescence/hour.

**Figure 1:**
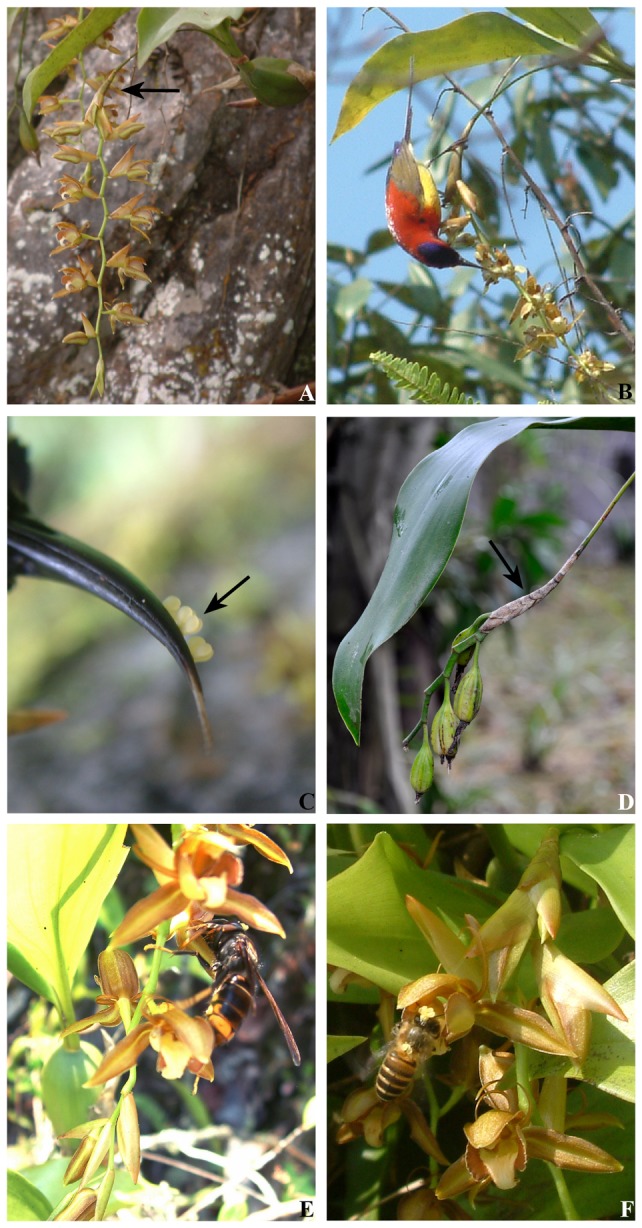
Sheaths surrounding the basal axis (perch) of *C. rigida* inflorescences attract and position sunbirds for cross-pollination to ensure reproductive success. **A.** Inflorescence with the sheathed bird perch (arrow). **B.** A bird (male) on a perch bends down to probe flowers. **C.** Pollinaria (arrow) attached to different spots on the bird beak are cross-transferred to flowers with equivalent distances to perch. **D.** Fruit set from cross-pollination. E. A wasp and F. a honeybee visit flowers in the same manner, both causing infertile self-pollination.

Sunbirds are active during daytime (07: 00 to 19: 30) and are frequent visitors of *C. rigida* flowers. When foraging, the male birds usually reach the flowering plants first, followed by the female birds. We observed that the birds perch only on the sheath-covered basal axis of the inflorescences during the process of nectar probing. When the sunbirds land on the sheaths, they reach for the nectar by laterally inserting their beaks into the base of the lip (Figs. 1B and S3). The birds were consistently observed to fly away immediately to find food in a neighboring population after they finish visiting an inflorescence. Of the 217 visits observed, all of the sunbirds probed for nectar and then left to search for food in another population. On the other hand, none of the birds observed visited inflorescences within the same plant clone. Female sunbirds spent 2.60 s ±0.54 s (n = 30) in visiting one inflorescence, whereas the male sunbirds spent 2.06 s ±0.34 s (n = 30) visiting one inflorescence. The results of the observations on the visiting behavior of the birds are presented in Tables S1 and S3.

The inflorescence of *C. rigida* is pendulous, its rachis is soft, and its flowers are below the basal axis and are open horizontally (Fig. S2). Consequently, the sunbirds perching on the basal axis have to bend their heads down to probe the flowers for nectar. The sheathed basal axis (perch) is 3 cm to 4 cm long and gradually enlarges toward the apex below (Fig. S2, inset), which is adequate for a sunbird to land and grasp onto the perch securely without slipping off when leaning downward (Figs. 1B and S3). A sunbird grasps the perch by using its feet upon landing and then leans down to feed. As the curved column and the base-saccate disc fuse into an arching “floral tube,” the linear petals bend backward and prepare a passage for sunbird visitation. When the sunbird laterally inserts its curved beak into the disc for nectar and then backs out, its beak touches the short ligulate rostellum, drawing the pollinarium. To reach and feed on the different flowers on the same inflorescence, the bird has to extend its neck and beak at various lengths and angles, thereby loading the pollinaria at different spots on its beak (Fig. 1C), which prevents self-pollen transfer among the flowers within the same inflorescence. When the bird visits the flowers of another plant's inflorescence, the pollinaria on the bird's beak are separately scraped into the stigma cavities by the rostella of these flowers. Then, the beak draws the pollinaria according to the positions and distances of the flowers relative to the bird perches, thereby completing the pollen transfer between plants. Once the stigma of the *C. rigida* accepts a pollinarium, its rostellum moves inward and covers the stigma cavity, which protects pollen development and fertilization inside and prevents the reception of additional pollen, thus yielding a fruit set (Fig. 1D). Among the eight captured sunbirds (five males and three females), the pollinaria of *C. rigida* were found to be attached to all their beaks (Fig. 1C). Each of the males weighed ca. 22 g, whereas the females weighed ca. 18 g.

The wasps (Fig. 1E) and honeybees (Fig. 1F) exhibited the same visitation behavior when visiting *C. rigida*. A foraging wasp or bee lands on the epichile of the lip and then climbs into the disc for nectar. While backing out, the insect touches the rostellum with its forehead and brings out the pollinarium. Then, the insect tends to visit another flower within the same inflorescence or plant clone. The insect then delivers the cargo pollinarium when probing this flower and draws its pollinarium when backing out, thereby resulting in self pollen transfer between flowers (Figs. 1E and 1F). During our observations of the 30 sites included in this study, the wasps and honeybees were found to have visited the flowers successfully. All wasps and honeybees were discovered to have continuously visited multiple flowers within the same inflorescences and within the same plant clone. The insects rarely flew away to visit flowers in other clones. The wasps spent 193.64 s ±70.36 s in visiting 27.80±13.72 inflorescences, whereas the honeybees spent 418.84 s ±159.39 s in visiting 62.23±22.81 inflorescences (Table S1).

### Floral odor analysis

The aromatic molecules detected in the fresh flowers of *C. rigida* through the TRACE gas chromatography with mass spectrometry (GC-MS) method comprised a combination of a sweet floral fragrance of pheol, 2,2′-methylenebis[6-(1,1-dimethylethyl)-4-methyl-], acetic acid, octadecyl ester, hexadecanamide, and *n*-hexadecanoic acid (Fig. S4).

### Measurement of nectar volume and sugar content

A bagged flower was observed to produce 10.03 µl ±1.02 µl (n = 20) of nectar with 20.80%±1.20% (n = 20) sugar content each day, whereas an open flower produced 9.98 µl ±1.08 µl (n = 20) of nectar with 21.00%±1.12% (n = 20) sugar content each day. The volume and production rates of nectar were similar in the morning and afternoon. There was no significant difference in nectar volume and sugar concentration between bagged flowers and open flowers, respectively (*t* = 0.1504, *d.f*. = 38, *P* = 0.8812; *t* = 0.5449, *d.f*. = 38, *P* = 0.5890), because the visiting rate was very low to open flowers, less than one visit (0.84 time)/flower (inflorescence)/three hours.

### Tests on the mating system, natural pollination, and bagged treatment

**Figure 2:**
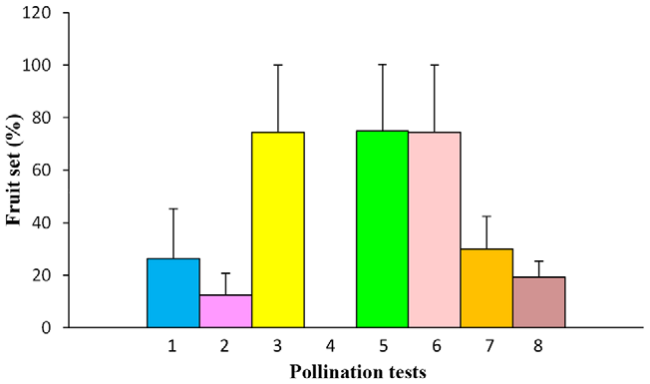
Fruit set rates of different pollination tests (**mean ± s.d., n = 30**)**:** **1.** In natural condition, with sheaths. **2.** In natural condition, removed sheaths. **3.** Artificial cross-pollination. **4.** Artificial self-pollination. **5.** Artificial cross-pollination, with sheaths. **6.** Artificial cross-pollination, removed sheaths. **7.** In natural condition, pollen removed. **8.** In natural condition, without any treatment.

The results of our tests on the mating system of *C. rigida* are shown in Table S2. The rate of fruit set of this species was 26.33%±19.06% (*n* = 30) in a natural environment and 74.22%±25.62% (*n* = 30) by artificial cross-pollination. However, the rate was 0 (*n* = 30) through artificial self-pollination, which shows that *C. rigida* is truly self-incompatible. The rate of fruit set of bagged flowers, unpollinated or artificially self-pollinated, was zero (*n* = 30), which indicates that the orchid is incapable of producing asexual seeds or of spontaneous autogamy. These results clearly show that *C. rigida* produces seeds only by crossing and that the observed natural fruit set (26.33%±19.06%) must have resulted from cross-pollination (Figure 2).

### Tests on sheath function

The fruit set of artificial cross-pollination (Table S2) was 74.73%±25.38% (*n* = 30) for inflorescences removed sheaths and 74.22%±25.62% (*n* = 30) for those with sheaths. No difference (*t* = 0.0769, *d.f*. = 58, *P* = 0.9390) was observed between these fruit set rates, and no change in floral morphology was observed after removing the sheaths, which indicates that sheath removal did not affect the floral development or the inherent capability of the *C. rigida* to set seeds or to promote seed growth. Evidently, the brown, coriaceous, and non-chlorophyllic sheaths are not a significant source of photosynthetic carbon for seed development, thus excluding any possible physiological function of the sheaths.

Sheath removal reduced the natural fruit set of *C. rigida* (Table S2). The inflorescences with sheaths had a natural fruit set rate of 26.33%±19.06% (*n* = 30), whereas those without sheaths had a natural fruit set rate of 12.30%±8.26% (*n* = 30), which resulted in significant differences (*t* = −3.6990, *d.f*. = 58, *P* = 0.0005). A greater than 53% drop in fruit set was observed after the sheaths were peeled off, which compromised but did not totally destroy the perch, as indicated by the intact inner part of the axis. This remarkable observation was clearly caused by a marked decrease in the frequency and duration of the visits of sunbirds. Among the sample pairs, 64 visits observed to have been made to inflorescences with sheaths that were removed, whereas 93 visits were found to be made to inflorescences with intact sheaths during the same period (Table S3). The male and female birds showed similar strong preferences for inflorescences with intact perches. The males made 51 visits to inflorescences with sheaths and 35 visits to those without sheaths, whereas the females made 42 visits to inflorescences with sheaths and 29 visits to those without sheaths. Moreover, both male and female birds spent longer periods of time visiting inflorescences with sheaths than those without sheaths. The time spent foraging by male birds was as follows: intact sheaths, 1.83 s ±0.41 s (*n* = 51), and sheaths that were removed, 1.46 s ±0.58 s (*n* = 35). Meanwhile, time spent foraging by female birds was as follows: intact sheaths, 2.67 s ±1.22 s (*n* = 42), and sheaths that were removed, 1.67 s ±0.58 s (*n* = 29). Significant differences in the duration of visits of male and female birds were observed between the inflorescences with sheaths and those without sheaths (Wilcoxon: Z = −3.1713, *d.f*. = 84, *P* = 0.00152; Z = −4.9980, *d.f*. = 69, *P* = 5.79349E–07). Finally, the male birds made appreciably more frequent visits to flowers, whereas the visits of female birds lasted longer.

**Figure 3:**
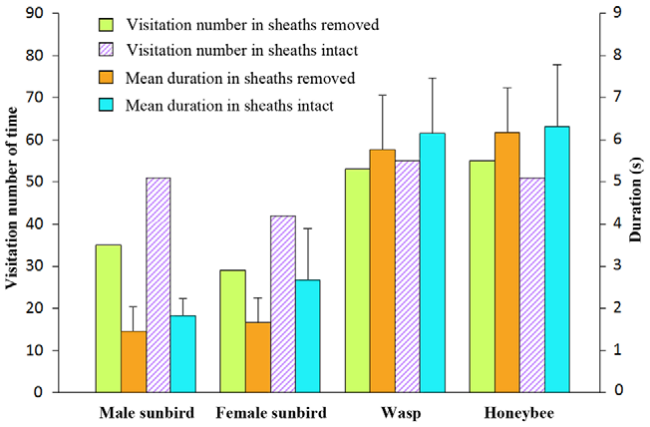
Visiting number of times and mean duration of pollinators between inflorescences with sheaths removed and with sheaths intact.

The visitations of *C. rigida* by insects were unaffected by the removal of sheaths. Wasps and honeybees visited the inflorescences at almost the same frequency with or without sheaths (55 vs. 53 times; 51 vs. 55 times). The wasps took 6.15 s ±1.31 s (*n* = 55) to visit inflorescences with sheaths and 5.76 s ±1.29 s (*n* = 53) to visit those without sheaths. These results are not significantly different (Wilcoxon: Z = −1.8707, *d.f*. = 106, *P* = 0.061394). The honeybees took 6.31 s ±1.47 s (*n* = 51) to visit inflorescences with sheaths and 6.18 s ±1.05 s (*n* = 55) to visit those without sheaths. Likewise, these results are not significantly different (Wilcoxon: Z = 0.52048, *d.f*. = 104, *P* = 0.60273) (Table S3; Figure 3).

### Tests on self-pollination: effect of pollen removal on fruit set

According to our observation, a flower generally withers five to seven days after the removal of its pollinaria. The natural levels of fruit set for plants with pollen removed from the entire inflorescence (Table S4) were 30.53%±12.26% (*n* = 30) and 19.30%±5.96% (*n* = 30) for those without any treatment. A significant difference (*t* = 4.5156, *d.f*. = 58, *P* = 3.15×10^−5^) was observed between the two groups. Self-pollen removal significantly enhanced the mating success of cross-pollination, which indicates that between-flower self-pollination (geitonogamy) indeed occurs in *C. rigida* (Figure 3). In the flowers that received self pollen from the same inflorescence or clone, the rostellum was found to cover the stigma cavity to prevent the flower from accepting foreign pollen after its own pollen entered the stigma. Self-incompatibility caused the termination of flower development, which wasted ovules and pollen that could have been used for cross-pollination.

### Pollination observations and mating system tests on *Coelogyne fimbriata*

*C. fimbriata*, a species related to *C. rigida* in all aspects except for the absence of a sheathed perch and bird pollination, is pollinated only by the wasp and bee species that also pollinate *C. rigida*. The insects exhibited the same pollination behavior, visiting frequency, and duration in *C. fimbriata* as in *C. rigida*. The fruit set rate of *C. fimbriata* is 0 (*n* = 20) from artificial self-pollination, which shows that it is self-incompatible, and 72.75%±10.57% (*n* = 20) from artificial cross-pollination. Both values are equivalent to those of *C. rigida*. Importantly, the fruit set rate of *C. fimbriata* in its natural environment is merely 0.50%±1.54% (*n* = 20), which indicates that insect pollination is almost entirely infertile selfing, with minimal fruitful crossing (Table S5), which is also probably true in *C. rigida*.

## Discussion

This study describes a new and perhaps a most striking example of a structural adaptation that promotes cross-pollination in angiosperms. The results show that the sheaths surrounding the basal axis of the inflorescence of *C. rigida*, a self-incompatible orchid, constitute a perch for attracting and positioning foraging sunbirds to conduct efficient and orderly cross-pollination, which is responsible for essentially all the seed production of *C. rigida*, to ensure its reproductive success. Simultaneously, *C. rigida* offsets insect-mediated self-pollination, which causes infertility and incurs mating cost, through gamete discounting. Consequently, *C. rigida* gains not only mating and fertility advantages and genetic variability from crossing, but more importantly, it also endures reproductive success through subtle structural adaptation by merely adding a perch to the basal axis instead of altering the inflorescences of multiple flowers. The development of the structure for bird cross-pollination is likely an evolutionary response to self-pollination by insects, which is rendered infertile by self-incompatibility and incurs a high mating cost, as well as to the lack of cross-pollination from insects.

Except for its discernible scent and prominent sheaths, *C. rigida* does not conform to the general description of bird-pollinated plants because its flowers are not brightly colored. The reward offered to pollinators is nectar, with sugar content that is lower in this species than in other insect-pollinated species because birds cannot sip nectar that is excessively viscous [12].

All the sunbirds we observed alighted on the coriaceous sheaths, which cover the basal axis of inflorescence, before probing the flowers for nectar. Given that the inflorescence of *C. rigida* is pendent with flowers horizontally opening along the soft axis, sunbirds must bend their heads to reach the nectar in the flowers. The sheathed perch is located at the base of the inflorescence directly above the flowers and has a conical shape. The sheath serves as an alighting perch and a “grasping pole” that is sufficient for the sunbirds to land and to grasp securely. Upon landing, sunbirds grasp the perch by using their feet. The birds then lean down to access nectar by inserting their curved beaks into the disc from one side of the flower. In this way, the floral stigma and the pollinarium of *C. rigida* successively come in contact with the bird's beak, enabling the pollinaria to be accepted by the bird and to be extracted from the flowers. Considering the confined standing position, sunbirds bend their bodies and elongate their tongues to visit different flowers with the same inflorescence. Different parts of their beaks, rather than their tongues, touch different floral columns and receive pollinaria. Thus, the pollinaria can only be delivered by the sunbird separately to the flowers of another inflorescence with corresponding distances between bird perch and flowers. Considering that the distances between a sunbird and different flowers are dissimilar and that the bird probes the flowers for nectar in a regular sequence, the pollinaria will not overlap with one another on the bird's beak or will not mix in the flowers, thereby avoiding self-pollination. Sunbirds do not revisit flowers within the same inflorescence or the same clone, thereby also avoiding selfing among those flowers and ensuring the successful transfer of pollen among different plant clones.

Experiments with perch cover removal (peeling off of the sheaths around axis) immediately before blooming demonstrate that the sheaths are not required for floral or seed development but are important factors in attracting and keeping the visiting sunbirds in position for cross-pollination. Sheath removal did not preclude visitations by sunbirds because the remaining bare axis could still serve as a damaged perch, but it markedly reduced the frequency and duration of bird visitations and, consequently, the fruit setting rate of the orchid. The sunbirds strongly prefer landing and feeding on inflorescences with intact perches than on those with damaged perches. The sheaths function as a signal for sunbirds to visit the flowers. Intact perches are constructed in such a way that helps sunbirds complete flower visitation, thus increasing pollination efficiency. Our observations show that an axis tightly covered by coriaceous sheaths at the inflorescence base is 3 cm to 4 cm long, with its lower part enlarged, vertically forming a narrow, conical perch that signals sunbirds to land and provides them with visiting perches. The sheathed perches help sunbirds grasp tightly and avoid slipping, which allows them access to flowers in the lower part of the inflorescence. If a sunbird heavier than 18 g lands directly on the flowers, as some bird species do [13], [14], the soft rachis and fragile flowers of *C. rigida* will not be capable of supporting its weight. Therefore, the stable sheaths guarantee sunbirds sufficient time to alight on a firm foothold until they have finished probing all open flowers on the inflorescence. Particularly, when probing flowers on the far end, the birds spend longer times hanging on the perch. The sheathed perch is an important factor in attracting birds and controlling their visiting positions to enable them to pick up and deliver pollen using different parts of their beaks. Thus, once the sheaths are removed, bird visitation decreases, which limits cross-pollination and fruit setting.

Although *C. rigida* visitations by male and female sunbirds were equally affected by perch cover removal, notable differences were observed in the visiting behavior of male and female birds. The male birds, which are distinguishable from the female birds by their size and color, seem more diligent in foraging and are more alert. On average, the males visited the orchid populations more frequently but stayed for a shorter time per visit. For couples of visiting birds, the males usually reached flowering populations earlier than females. Males may need more food because they are larger and have a greater responsibility to hunt for food when females are nesting. For males, staying on the perches to probe flowers could be less convenient because of their longer tail feathers and greater weight. Furthermore, their colorful feathers could make them more vulnerable to predation if they stay at one site for too long.

We also examined the function of the floral lip, the other landing plate for visiting insect pollinators. Similar to sunbirds, wasps and honeybees are attracted by the nectar of the *C. rigida* flower. Both insects land on the epichile of the lip and then crawl into the disc to probe for nectar. While backing out, the forehead of wasps and honeybees come into contact with the rostellum, which facilitates pollen transfer. Considering that insects tend to visit flowers on the same inflorescence and in the same plant clone continuously and repeatedly, their pollen transfer is virtually all self-pollination, which results in abortive fertilization because of self-incompatibility, as well as in the wasting of pollen and ovules (gamete discounting) that could be used for fruitful cross-pollination.

*C. rigida* often forms a large number of plant clones, with 100 to 300 inflorescences opening simultaneously. We compared birds and insects that alight on different landing plates and found that sunbirds visit only one inflorescence in a clone before flying to another clone (a different plant), whereas wasps and honeybees visit different flowers of the same inflorescence or plant repeatedly for 3 min to 8 min or even longer to visit a clone, which results in self-pollination within an inflorescence or a clone. Pollen used for infertile selfing rather than fruitful outcrossing reduces usable pollen and male fitness as well as usable ovules and female fitness because of the lack of seeds from ovules. This behavior is unfavorable to *C. rigida* breeding. The natural fruit setting of inflorescences with all their pollinaria removed is significantly higher than that of unmanipulated inflorescences, which indicates that fruitless self-pollination occurs in *C. rigida* and is unavoidable because of visitation by insects. The insects repeatedly and continuously visit an inflorescence and then transfer their pollen to flowers of the same plant clone. This behavior diminishes the pollen available for pollinating other clones and the stigmas available for accepting foreign pollen, thereby contributing to pollen and ovule discounting. Removing the pollen of inflorescences offsets ovule discounting by enabling stigmas to accept foreign pollen, thus resulting in an increase in fruit set.

Under natural conditions, the contribution made by insects to the cross-pollination of *C. rigida* is difficult to quantify experimentally. Given that the insects rarely visit flowers in another plant clone, cross-pollination by the insects must be very low. Furthermore, *C. fimbriata*, an orchid related to *C. rigida*, has mostly the same characteristics, including growth in the same habitat with numerous clones, self-incompatibility, and the same insect pollinators except for birds because of the absence of the sheathed perch, i.e., it is exclusively self-pollinated by insects. Thus, *C. fimbriata* can be used as a suitable reference and control for *C. rigida* in studies on sheaths and pollination by insects and birds. We determined that although the fruit setting rate from the artificial cross-pollination of *C. fimbriata* (72.75%) was equivalent to that of *C. rigida*, the natural fruit setting rate of *C. fimbriata*, which exclusively results from cross-pollination by insects because selfing is infertile, was evidently low at 0.5%, as previously described [15]. Thus, in natural *C. rigida* populations, cross-pollination by insects more likely results in extremely low levels of fruit setting, similar to that in *C. fimbriata*. The remaining bulk of the total natural fruit set in *C. rigida* (26.33%) is likely contributed by cross-pollination by sunbirds, which indicates that bird perch-enabled cross-pollination is responsible for essentially all instances of sexual reproductive success of *C. rigida*.

In other words, if *C. rigida* did not develop the bird perch or if its bird perches were all eliminated, its cross-pollination and natural fruit setting would be extremely low, as found in *C. fimbriata*. Conversely, if *C. fimbriata* added such a perch for bird cross-pollination, its natural cross-pollination and natural fruit setting would have been significantly higher as in the case of *C. rigida*. Self-incompatibility is generally accompanied by an adaptive change in floral components to prevent self-pollination. Neither of these two species evolved such an adaptive mechanism to avoid self-pollination directly. However, *C. rigida* has developed a bird perch to promote cross-pollination directly and to offset self-pollination by insects, a feat unachieved by *C. fimbriata*.

The evolution of such a seemingly simple structural adaptation in *C. rigida*, which added sheaths around the flower-bearing basal axis instead of altering the inflorescence of many flowers, is remarkable. The development of such an optimal perch for attracting sunbirds and precisely positioning them for efficient and orderly pollen dispersal for cross-pollination to ensure reproductive success and to reduce mating costs is more striking and delicate than the previously reported case of *Babiana ringens*. For *B. ringens*, a self-compatible iris with a stand-alone perch enhances cross-pollination by sunbirds while the plant also reproduces via self-pollination by the birds [16]. However, the underlying mechanism is unclear, and cross-pollination is not required for the reproduction of the species.

With self-incompatibility, *C. rigida* requires a cross-pollination mechanism for successful sexual reproduction. The sheathed perch-enabled cross-pollination by sunbirds in *C. rigida* is ingenious and advantageous. Compared with changing the design of multiple individual flowers on each inflorescence, making one perch in each inflorescence (i.e., treating an inflorescence as an organized unit) is significantly more economical and enables more orderly control of pollination. Sunbirds are highly alert and active in a wide area, move rapidly, and spend a long time foraging to meet their large food requirements [12]. The perches on the *C. rigida* inflorescences confine sunbird movements to ensure the attachment of pollinaria to different parts of their beaks for cross-pollination. Pollinators such as sunbirds enable efficient and orderly pollen transfer between different plants to achieve cross-pollination, which also counteracts infertile self-pollination by insects to reduce genetic costs. Consequently, *C. rigida* does not need to modify its floral component to prevent self-pollination or to promote cross-pollination.

A basic characteristic of orchids is a specialized lip that is suitable for insect visitation [17]. Although *C. rigida* occupies a more recently evolved position [2] in the evolution of orchids, its lip is inherited from its ancestors and may have been derived from adaptation to pollination by bees. The lip of *C. rigida* (Epidendroideae) may share the same history with that of other species in the subfamily Orchidoideae. These clues are helpful in understanding the evolutionary implications of the bird-pollination mechanism to the plant breeding system.

*C. rigida* provides a uniquely interesting example in which the self-pollination mode complements the cross-pollination mode but is rendered infertile by a self-incompatible genetic mechanism. Self-pollination by insects is aided by the floral lip, whereas cross-pollination by birds is facilitated by the sheathed perch. The species likely reproduce through insect-mediated self-pollination, but the resultant inbreeding depression facilitated the evolution of self-incompatibility (to avoid inbreeding). Self-incompatibility necessitates and favors the development of an outbreeding mechanism, an example of which is the perch-facilitated cross-pollination by birds, to ensure reproductive success while reducing the gamete discounting (mating cost or waste) of self-pollination. The results and analysis suggest that in *C. rigida*, the mechanism of self-incompatibility may have evolved from that of self-compatibility, its outcrossing may have originated from selfing, and its bird-pollination mechanism may have evolved more recently. This finding would be consistent with the hypothesis that selfing is part of a larger process that promotes outcrossing [18], [19] or, at least, that the two pollination modes can develop into each other.

Recently, conflicting selection of floral traits by different pollinators has been thought to be important in the evolution of specialized species [20]–[23]. In *C. rigida*, bi-modal pollination systems coexist, wherein two types of visitors (birds and insects) can serve as pollinators, with birds strongly promoting cross-pollination and insects promoting geitonogamy. The selection forces acting on floral and inflorescence traits by pollinators must be closely related to the variation of the traits selected and to the plant reproductive success rate. *C. rigida* would probably develop an efficient variation of floral traits to prevent self-pollination caused by insect visits because auto-pollination is useless for a self-incompatible plant. However, ensuring the successful reproduction of *C. rigida* is a “task” of top priority, which has been fulfilled by its sheathed perch and special pollinator, the sunbird. *C. rigida* may require more time to change its floral traits to respond to new conditions.

Through the addition of sheaths around the axis of inflorescence to make a specialized perch that attracts and positions foraging sunbirds for orderly cross-pollination, *C. rigida* gains mating and fertility advantages and genetic variability. More importantly, the structure ensures reproductive success. This situation provides a new and striking example of a structural (non-floral) adaptation that promotes cross-pollination in angiosperms. This structural adaptation may shed light on the evolution of multi-flowered inflorescences in a large number of plants. Furthermore, the adaptation of inflorescence structure for bird pollination may represent an evolutionary trend in *Coelogyne*. Similar sheaths occur in other *Coelogyne* species, especially in the sections *Elatae* and *Proliferae*. The sheaths are found in all members of these two sections at the base of a pendent inflorescence or the apex of an erect one, i.e., the potential bird perches are invariably situated above all flowers of the inflorescence probably to facilitate pollen dispersal by birds effectively, as in *C. rigida*. Thus, our findings on *C. rigida* as a model may have broad implications for the evolution of flowering plants, particularly those with multi-flowered inflorescences, and their mating systems and strategies.

## Materials and Methods

From March to May of 2008, 2009, and 2010, the flowers and pollination biology of *C. rigida* were observed in an evergreen broad-leaved forest on a limestone slope at elevations ranging from 1500 m to 1800 m in Southeastern Yunnan, China. Up to 30 populations were chosen for this study, all of which are located in the said low-mountain region, having a subtropical plateau monsoon climate [24]. The forests in the region are dense.

All necessary permits were obtained for our field studies. The locations for our field studies were not private lands but protected areas. Our field observations did not collect any plant, animal, or insect specimen.

### Morphologic observation

The vegetative and floral features were observed in the Huoshaoliangzi Nature Reserve in Malipo, Southeast Yunnan. A total of 10 flowering plants were transplanted into the nursery of the National Orchid Conservation Center of China in Shenzhen. Fresh flowers were collected and observed directly under a stereomicroscope (Guiguang XTL-500, Guilin, China) to examine the structure of the perianth, column, and ovary.

### Pollination observation

Up to 20 inflorescences were randomly selected from 10 populations annually, that is, two inflorescences per population each year. The selected flowers were marked and continuously observed for five days under natural conditions each year. The species and number of visitors (birds and insects) for each marked inflorescence (flower) was observed continuously from 06∶00 to 20∶00 daily, and their visiting behavior was photographed and described. The following items were recorded for 10 visitation of each visitor every year: the number of inflorescence for each visitation, the frequency of visitation to one flower, the time of a visitor spent on an inflorescence, and the number of flowers visited. Visitors touched anther or stigma were collected for further identification and examination.

### Floral odor analysis

Five flowering *C. rigida* plants were randomly selected from five populations, which were then potted and sent to the laboratory of South China Agricultural University in Guangzhou. Five fresh, unpollinated flowers were cut off from a plant to collect odor samples. These samples were placed in 100 ml headspace bottles. Meanwhile, 75 μm of carboxen-polydimethylsiloxane was extracted (30 min), desorbed (3 min, 200°C), and analyzed on a Finnigan TRACE GC/MS (25°C, 65% humidity). The volatile components were analyzed at the Analysis Center of South China Agricultural University.

### Measurement of nectar volume and sugar content

The volumes of nectar from 20 flowers bagged before opening and 20 flowers unbagged before opening were measured using a 5 µl to 10 µl micropipette every three hours from 6∶00 to 20∶00. If nectar was detected, the sugar content (%) was directly measured with a handheld refractometer (Taiguang 409122, Chengdu, China).

### Tests on mating system

A total of 60 sites were randomly selected for a controlled test of artificial self-pollination (30 sites) and artificial cross-pollination (30 sites) for three consecutive years from 2008 to 2010. Up to 20 sites were chosen annually, with each site having eight flowers to 10 flowers.

#### Artificial self-pollination

All flowers tested were bagged before blooming. After blooming but before fertilization, the bags were opened temporarily, and the pollinaria of the flowers were peeled off and placed into its own stigma cavity. Thereafter, the flowers were again immediately bagged. The changes in the flowers and the state of fruit setting were recorded.

#### Artificial cross-pollination

Flowers from the paired plants at the same site were bagged before blooming. After blooming but before fertilization, the bags were opened temporarily, and the pollinarium of one flower was peeled off and placed in the stigma cavity of another flower of a different plant, and vice versa. As soon as the flowers were pollinated, they were again bagged. The changes in the flowers and the state of fruit setting were recorded.

### Natural pollination and bagged treatment

A total of 60 sites were randomly selected from 2008 to 2010 for a controlled test of natural pollination (the flowers were not manipulated) and bagged treatment (the soon-to-bloom flowers were enclosed with a transparent bag to prevent the entry of insects). Each treatment had 10 sites annually with eight flowers to 10 flowers at each site. The states of pollination and fruit setting were observed and recorded.

### Tests on the function of sheaths around the axis

A total of 10 sample pairs were set up. Each sample had two inflorescences, one of which had its sheaths removed. Hidden in the bushes approximately 3 m away from the inflorescence, we observed and recorded the following data: number of visitors (birds and insects) of each inflorescence, time spent by a visitor on a flower, number of flowers visited by one visitor, and time spent lingering by a visitor in a population. To test the effect of sheath removal on fruit setting, 10 sample pairs were set up annually and were bagged before anthesis. The sheaths were removed from half of the inflorescences in each sample pair after blooming. All flowers were bagged after artificial cross-pollination (outbreeding), and the fruit setting rates of the two treatments were calculated.

### Mating system tests and pollination observations on *C. fimbriata*

Mating system tests and pollination observations on *C. fimbriata* were conducted from 2008 to 2009. The differences in the pollination effects between *C. fimbriata* and *C. rigida* were compared.

### Detection of self-pollination: effect of pollen removal on fruit setting

Two sample pairs were set up in each of 10 populations yearly from 2008 to 2010. Each sample included two inflorescences that were bagged at the bud stage until all flowers bloomed. All pollen was removed from one of the two inflorescences. Flowers were then visited by pollinators in a natural environment. Both natural fruit sets were counted after anthesis.

## Supporting Information

Figure S1 **Large number of plant clones of** ***C. rigida*** **with flowers on numerous inflorescences opening simultaneously.**
(TIF)

Figure S2 **Multi-flowered and pendent inflorescence of** ***C. rigida*** **, with a specialized bird perch made of sheaths around the basal axis** (**arrow**)**.**
(TIF)

Figure S3 **Female sunbird on the perch of** ***C. rigida*** **, leaning to probe flowers.**
(TIF)

Figure S4 **Gas chromatogram of the floral fragrance of** ***C. rigida*** **.**
(TIF)

Table S1 **Number of inflorescences and time**(**s**) **of each visit to a clone by a pollinator.**
(DOC)

Table S2 **Observation results of pollination experiments on the mating system of** ***C. rigida*** **.**
(DOC)

Table S3 **Time**(**s**) **of each visit to the inflorescence with sheaths and without sheaths.**
(DOC)

Table S4 **Rate of natural fruit setting of inflorescence with pollen removed and pollen present in** ***C. rigida*** **.**
(DOC)

Table S5 **Observation results of pollination experiments on the mating system of** ***C. fimbriatum*** **.**
(DOC)
