# Supplementary figures and images for: Adding Perches for Cross-Pollination Ensures the Reproduction of a Self-Incompatible Orchid
Source: PLoS One. 2013 Jan 7;8(1):e53695. doi: 10.1371/journal.pone.0053695 (PMC3538729; doi:10.1371/journal.pone.0053695)

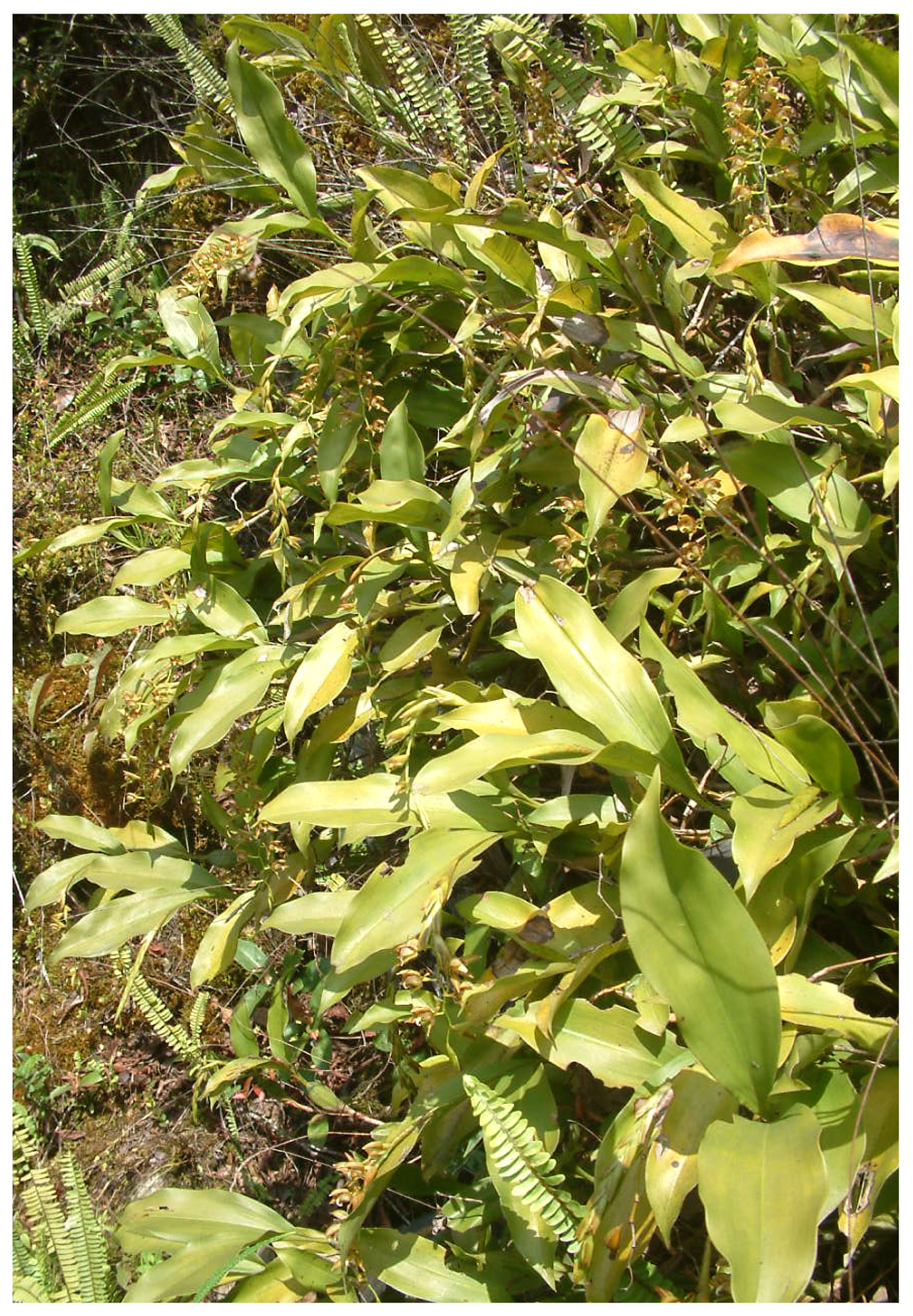

Supplement: Figure S1 — Large number of plant clones of C. rigida with flowers on numerous inflorescences opening simultaneously. (TIF) [file pone.0053695.s001.tif]

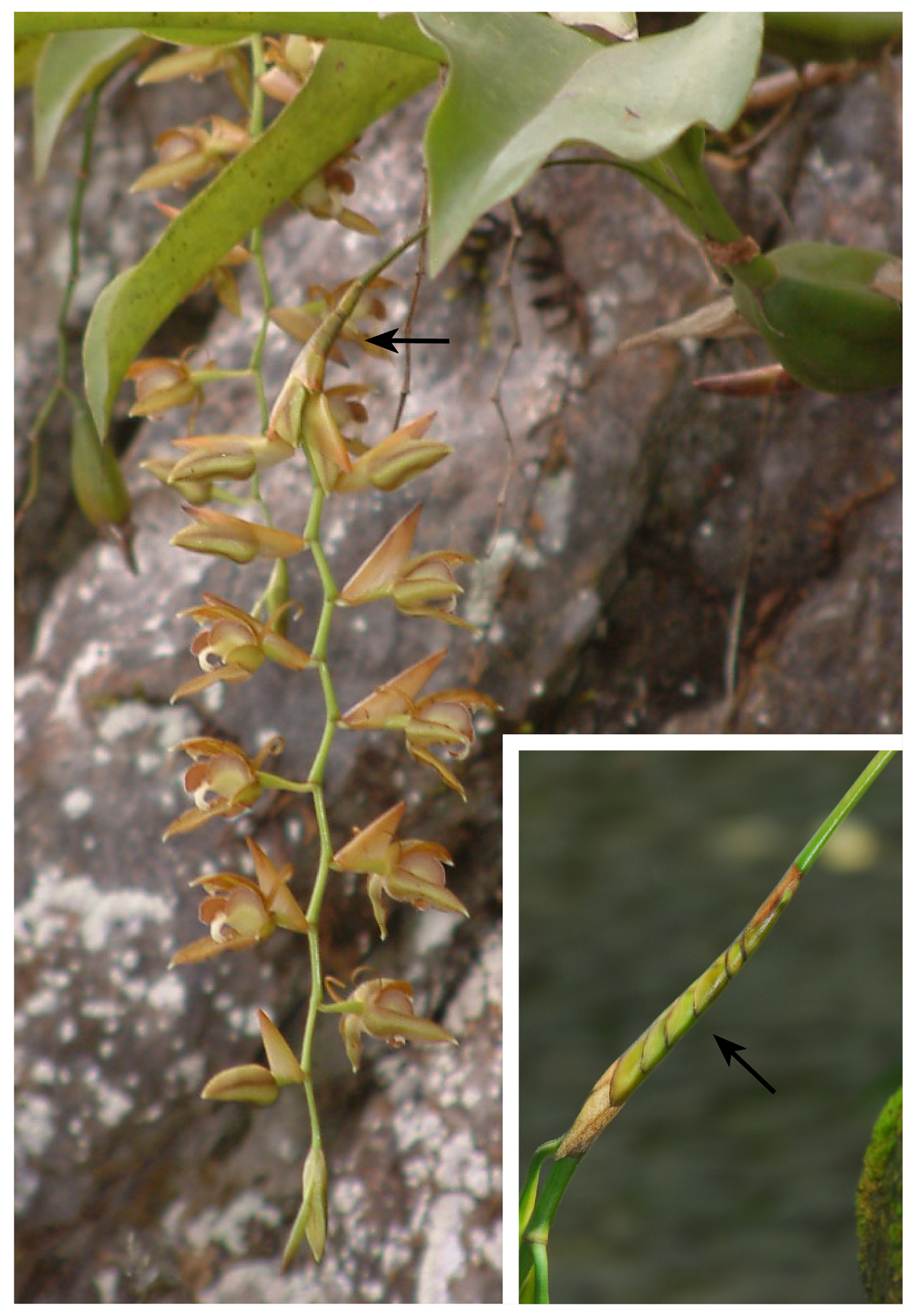

Supplement: Figure S2 — Multi-flowered and pendent inflorescence of C. rigida , with a specialized bird perch made of sheaths around the basal axis (arrow). (TIF) [file pone.0053695.s002.tif]

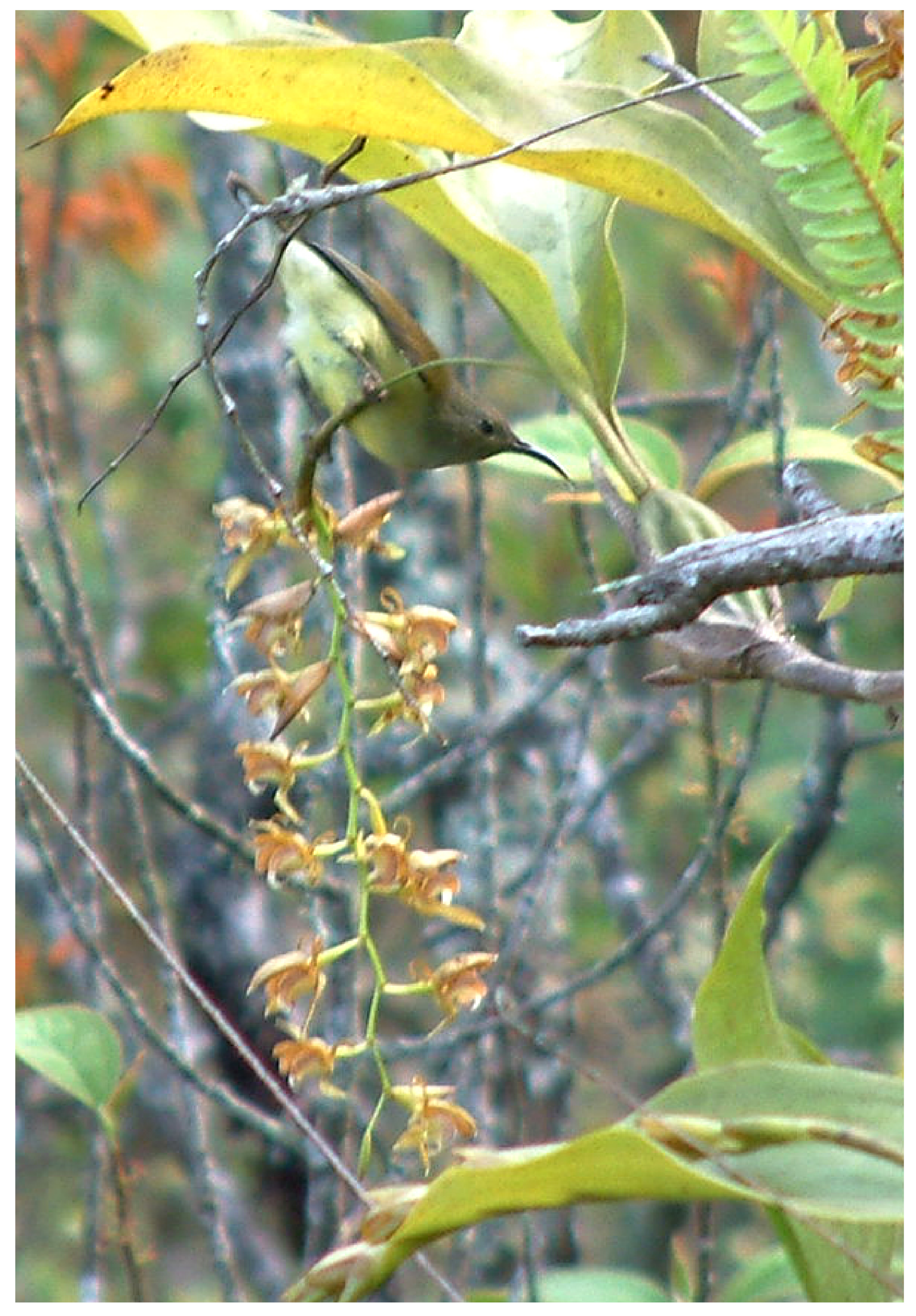

Supplement: Figure S3 — Female sunbird on the perch of C. rigida , leaning to probe flowers. (TIF) [file pone.0053695.s003.tif]

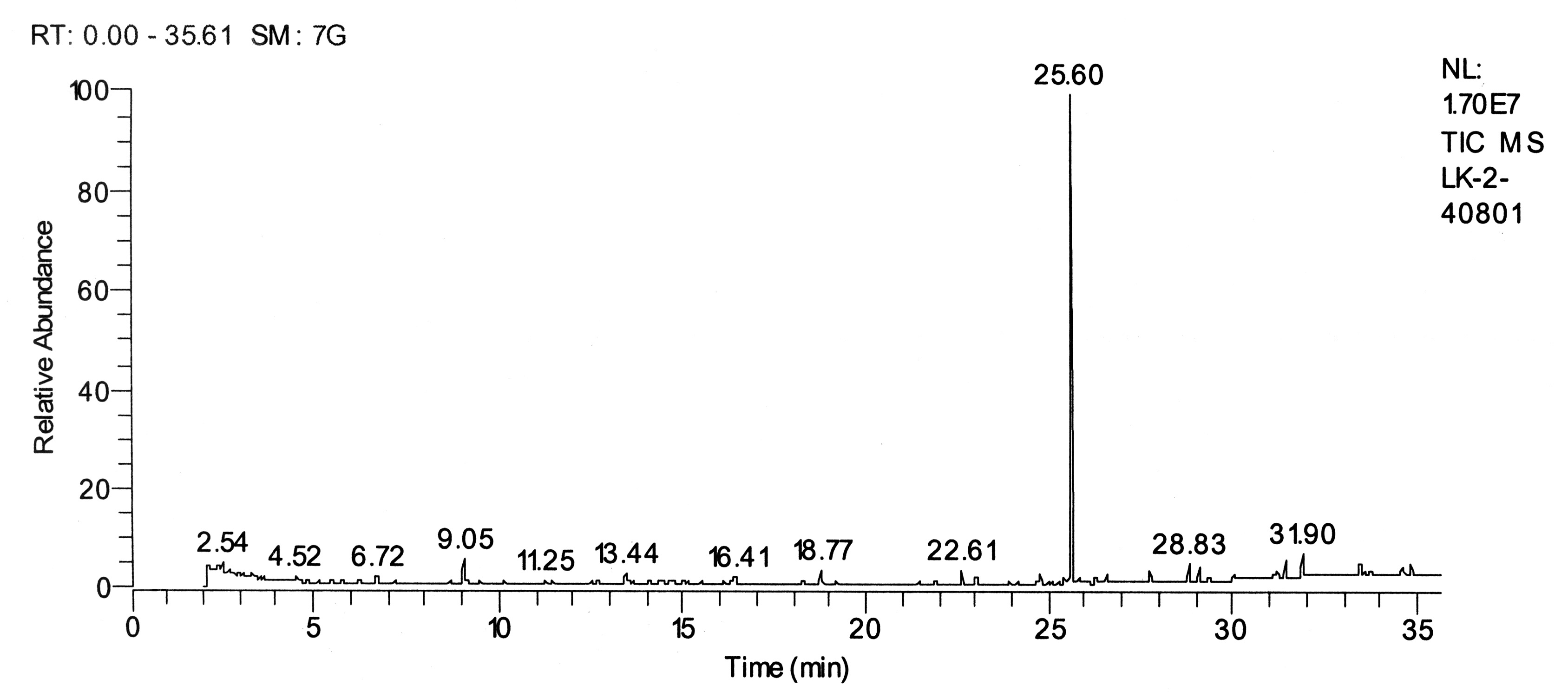

Supplement: Figure S4 — Gas chromatogram of the floral fragrance of C. rigida . (TIF) [file pone.0053695.s004.tif]
