# Supplementary material for: Adding Perches for Cross-Pollination Ensures the Reproduction of a Self-Incompatible Orchid
Source: PLoS One. 2013 Jan 7;8(1):e53695. doi: 10.1371/journal.pone.0053695 (PMC3538729; doi:10.1371/journal.pone.0053695)
Supplement: Table S1 — Number of inflorescences and time(s) of each visit to a clone by a pollinator. (DOC) [file pone.0053695.s005.doc]

**Table S1.** Number of inflorescences and time(s) of each visit to a clone by a pollinator

| Sample no. | Sunbird | | | | | Honeybee | | | | Wasp | |
| --- | --- | --- | --- | --- | --- | --- | --- | --- | --- | --- | --- |
|  | Female | | | Male | |  |  |  |  |  |  |
|  | No. of inflorescences | Time |  | No. of inflorescences | Time |  | No. of inflorescences | Time |  | No. of inflorescences | Time |
|  | 1 | 3.44 | | 1 | 1.49 | 46 | | 275.42 | | 33 | 181.73 |
|  | 1 | 2.42 | | 1 | 1.98 | 70 | | 350.53 | | 42 | 231.43 |
|  | 1 | 1.48 | | 1 | 2.13 | 102 | | 871.62 | | 19 | 119.57 |
|  | 1 | 2.41 | | 1 | 1.37 | 87 | | 431.87 | | 32 | 192.83 |
|  | 1 | 2.49 | | 1 | 2.08 | 68 | | 374.54 | | 26 | 178.01 |
|  | 1 | 2.47 | | 1 | 2.12 | 121 | | 820.82 | | 18 | 170.63 |
|  | 1 | 3.31 | | 1 | 2.32 | 37 | | 245.12 | | 71 | 429.68 |
|  | 1 | 2.46 | | 1 | 2.11 | 67 | | 388.71 | | 23 | 131.32 |
|  | 1 | 2.45 | | 1 | 2.03 | 92 | | 602.62 | | 31 | 181.92 |
|  | 1 | 3.12 | | 1 | 1.48 | 103 | | 716.57 | | 42 | 252.03 |
|  | 1 | 1.59 | | 1 | 1.38 | 38 | | 382.41 | | 14 | 168.56 |
|  | 1 | 3.33 | | 1 | 2.47 | 62 | | 412.38 | | 27 | 226.80 |
|  | 1 | 3.12 | | 1 | 1.90 | 70 | | 511.46 | | 32 | 252.00 |
|  | 1 | 3.01 | | 1 | 2.30 | 39 | | 352.67 | | 18 | 144.00 |
|  | 1 | 2.32 | | 1 | 1.97 | 42 | | 405.12 | | 61 | 329.40 |
|  | 1 | 2.58 | | 1 | 2.01 | 47 | | 387.90 | | 38 | 349.60 |
|  | 1 | 2.87 | | 1 | 2.10 | 79 | | 513.41 | | 31 | 163.71 |
|  | 1 | 3.01 | | 1 | 2.87 | 53 | | 303.44 | | 41 | 193.20 |
|  | 1 | 1.69 | | 1 | 1.72 | 68 | | 417.83 | | 25 | 156.71 |
|  | 1 | 2.34 | | 1 | 1.98 | 64 | | 392.86 | | 17 | 146.32 |
|  | 1 | 3.02 | | 1 | 2.56 | 58 | | 506.31 | | 15 | 161.17 |
|  | 1 | 2.89 | | 1 | 2.13 | 71 | | 492.11 | | 23 | 186.67 |
|  | 1 | 2.56 | | 1 | 1.98 | 36 | | 278.33 | | 20 | 177.63 |
|  | 1 | 2.67 | | 1 | 2.01 | 51 | | 312.30 | | 18 | 168.91 |
|  | 1 | 2.89 | | 1 | 2.30 | 47 | | 243.30 | | 21 | 170.62 |
|  | 1 | 3.11 | | 1 | 2.43 | 79 | | 391.32 | | 31 | 183.72 |
|  | 1 | 2.87 | | 1 | 2.56 | 63 | | 349.72 | | 16 | 150.62 |
|  | 1 | 2.80 | | 1 | 1.99 | 42 | | 361.84 | | 27 | 148.27 |
|  | 1 | 1.90 | | 1 | 2.01 | 28 | | 219.28 | | 13 | 172.52 |
|  | 1 | 1.52 | | 1 | 2.04 | 37 | | 253.40 | | 9 | 89.71 |
| $\bar{x}$ | 1 | 2.6 | | 1 | 2.06 | 62.23 | | 418.84 | | 27.8 | 193.64 |
| SD | 0 | 0.54 | | 0 | 0.34 | 22.81 | | 159.39 | | 13.72 | 70.36 |
