# Supplementary material for: Adding Perches for Cross-Pollination Ensures the Reproduction of a Self-Incompatible Orchid
Source: PLoS One. 2013 Jan 7;8(1):e53695. doi: 10.1371/journal.pone.0053695 (PMC3538729; doi:10.1371/journal.pone.0053695)
Supplement: Table S2 — Observation results of pollination experiments on the mating system of C. rigida . (DOC) [file pone.0053695.s006.doc]

***Table S2.*** *Observation results of pollination experiments on the mating system of C. rigida*

| Sample pair | Artificial cross-pollination | | | | | | | | Artificial self-pollination | | | | | Natural pollination | | | | | | | | | | |
| --- | --- | --- | --- | --- | --- | --- | --- | --- | --- | --- | --- | --- | --- | --- | --- | --- | --- | --- | --- | --- | --- | --- | --- | --- |
|  | Sheaths removed | | | | Sheaths present | | |  | Sheaths present | | | |  | Sheaths removed | | | | Sheaths present | | | | Bagged | | |
|  | No. of flowers | No. of capsules | Rate of fruit set |  | No. of flowers | No. of capsules | Rate of fruit set |  | | No. of flowers | No. of capsules | Rate of fruit set |  | No. of flowers | No. of capsules | Rate of fruit set |  | No. of flowers | No. of capsules | Rate of fruit set |  | No. of flowers | No. of capsules | Rate of fruit set |
|  | 14 | 12 | 85.71 | | 14 | 10 | 71.43 | | | 24 | 0 | 0 | | 18 | 0 | 0 | | 16 | 2 | 12.50 | | 18 | 0 | 0 |
|  | 10 | 8 | 80.00 | | 10 | 10 | 100 | | | 14 | 0 | 0 | | 18 | 4 | 22.22 | | 18 | 4 | 22.22 | | 19 | 0 | 0 |
|  | 18 | 18 | 100 | | 18 | 16 | 88.89 | | | 16 | 0 | 0 | | 16 | 2 | 12.50 | | 16 | 2 | 12.50 | | 20 | 0 | 0 |
|  | 16 | 12 | 75.00 | | 16 | 14 | 87.50 | | | 19 | 0 | 0 | | 12 | 2 | 16.67 | | 12 | 2 | 16.67 | | 20 | 0 | 0 |
|  | 18 | 14 | 77.78 | | 18 | 14 | 77.78 | | | 25 | 0 | 0 | | 12 | 0 | 0 | | 12 | 2 | 16.67 | | 17 | 0 | 0 |
|  | 16 | 16 | 100 | | 20 | 20 | 100 | | | 22 | 0 | 0 | | 10 | 2 | 20.00 | | 10 | 2 | 20.00 | | 30 | 0 | 0 |
|  | 20 | 18 | 90.00 | | 20 | 18 | 90.00 | | | 18 | 0 | 0 | | 18 | 4 | 22.22 | | 18 | 4 | 22.22 | | 24 | 0 | 0 |
|  | 30 | 28 | 93.33 | | 30 | 26 | 86.67 | | | 15 | 0 | 0 | | 20 | 2 | 10.00 | | 20 | 4 | 20.00 | | 22 | 0 | 0 |
|  | 24 | 20 | 83.33 | | 24 | 22 | 91.67 | | | 17 | 0 | 0 | | 10 | 3 | 30.00 | | 10 | 8 | 80.00 | | 21 | 0 | 0 |
|  | 16 | 14 | 87.50 | | 16 | 14 | 87.50 | | | 23 | 0 | 0 | | 12 | 2 | 16.67 | | 12 | 10 | 83.33 | | 20 | 0 | 0 |
|  | 14 | 4 | 28.57 | | 10 | 4 | 40.00 | | | 24 | 0 | 0 | | 16 | 2 | 12.50 | | 16 | 2 | 12.50 | | 19 | 0 | 0 |
|  | 10 | 4 | 40.00 | | 14 | 4 | 28.57 | | | 23 | 0 | 0 | | 18 | 0 | 0 | | 18 | 2 | 11.11 | | 19 | 0 | 0 |
|  | 18 | 18 | 100 | | 18 | 18 | 100 | | | 28 | 0 | 0 | | 26 | 2 | 7.69 | | 28 | 4 | 14.29 | | 23 | 0 | 0 |
|  | 16 | 2 | 12.50 | | 16 | 2 | 12.50 | | | 18 | 0 | 0 | | 22 | 2 | 9.09 | | 22 | 2 | 9.09 | | 25 | 0 | 0 |
|  | 18 | 4 | 22.22 | | 18 | 4 | 22.22 | | | 19 | 0 | 0 | | 30 | 4 | 13.33 | | 24 | 4 | 16.67 | | 22 | 0 | 0 |
|  | 18 | 18 | 100 | | 16 | 16 | 100 | | | 24 | 0 | 0 | | 30 | 5 | 16.67 | | 28 | 6 | 21.43 | | 17 | 0 | 0 |
|  | 14 | 12 | 85.71 | | 16 | 14 | 87.50 | | | 21 | 0 | 0 | | 24 | 2 | 8.33 | | 26 | 8 | 30.77 | | 16 | 0 | 0 |
|  | 18 | 16 | 88.89 | | 18 | 16 | 88.89 | | | 20 | 0 | 0 | | 26 | 4 | 15.38 | | 24 | 4 | 16.67 | | 15 | 0 | 0 |
|  | 28 | 26 | 92.86 | | 26 | 16 | 61.54 | | | 22 | 0 | 0 | | 16 | 2 | 12.50 | | 28 | 6 | 21.43 | | 19 | 0 | 0 |
|  | 26 | 20 | 76.92 | | 26 | 18 | 69.23 | | | 25 | 0 | 0 | | 18 | 4 | 22.22 | | 18 | 4 | 22.22 | | 20 | 0 | 0 |
|  | 17 | 15 | 88.24 | | 13 | 10 | 76.92 | | | 23 | 0 | 0 | | 20 | 3 | 15.00 | | 19 | 3 | 15.79 | | 16 | 0 | 0 |
|  | 21 | 17 | 80.95 | | 20 | 18 | 90.00 | | | 15 | 0 | 0 | | 13 | 0 | 0 | | 21 | 4 | 19.05 | | 19 | 0 | 0 |
|  | 24 | 20 | 83.33 | | 23 | 10 | 43.48 | | | 17 | 0 | 0 | | 15 | 2 | 13.33 | | 22 | 6 | 27.27 | | 21 | 0 | 0 |
|  | 23 | 10 | 43.48 | | 19 | 15 | 78.95 | | | 19 | 0 | 0 | | 17 | 1 | 5.88 | | 13 | 4 | 30.77 | | 28 | 0 | 0 |
|  | 18 | 8 | 44.44 | | 20 | 17 | 85.00 | | | 27 | 0 | 0 | | 21 | 3 | 14.29 | | 27 | 8 | 29.63 | | 17 | 0 | 0 |
|  | 13 | 11 | 84.62 | | 28 | 12 | 42.86 | | | 26 | 0 | 0 | | 28 | 2 | 7.14 | | 17 | 2 | 11.76 | | 15 | 0 | 0 |
|  | 12 | 4 | 33.33 | | 17 | 17 | 100.00 | | | 21 | 0 | 0 | | 26 | 2 | 7.69 | | 23 | 9 | 39.13 | | 18 | 0 | 0 |
|  | 27 | 23 | 85.19 | | 20 | 17 | 85.00 | | | 21 | 0 | 0 | | 14 | 0 | 0 | | 10 | 7 | 70.00 | | 22 | 0 | 0 |
|  | 24 | 22 | 91.67 | | 18 | 7 | 38.89 | | | 22 | 0 | 0 | | 10 | 3 | 30.00 | | 11 | 4 | 36.36 | | 23 | 0 | 0 |
|  | 22 | 19 | 86.36 | | 16 | 15 | 93.75 | | | 27 | 0 | 0 | | 26 | 2 | 7.69 | | 18 | 5 | 27.78 | | 20 | 0 | 0 |
| $\bar{x}$ | 18.77 | 14.43 | 74.73 | | 18.60 | 13.80 | 74.22 | | | 21.17 | 0 | 0 | | 18.73 | 2.20 | 12.30 | | 18.57 | 4.47 | 26.33 | | 20.17 | 0 | 0 |
| SD | 5.21 | 6.82 | 25.38 | | 4.75 | 5.61 | 25.62 | | | 3.84 | 0 | 0 | | 6.05 | 1.35 | 8.26 | | 5.77 | 2.36 | 19.06 | | 3.51 | 0 | 0 |
