# Supplementary material for: Adding Perches for Cross-Pollination Ensures the Reproduction of a Self-Incompatible Orchid
Source: PLoS One. 2013 Jan 7;8(1):e53695. doi: 10.1371/journal.pone.0053695 (PMC3538729; doi:10.1371/journal.pone.0053695)
Supplement: Table S3 — Time(s) of each visit to the inflorescence with sheaths and without sheaths. (DOC) [file pone.0053695.s007.doc]

**Table S3.** Time(s) of each visit to the inflorescence with sheaths and without sheaths

| Sample pair | Sheaths present | | | | | | Sheaths removed | | | | | | | |
| --- | --- | --- | --- | --- | --- | --- | --- | --- | --- | --- | --- | --- | --- | --- |
|  | Sunbird | | | Wasp | Honeybee |  | | Sunbird | | | Wasp | | Honeybee | |
|  | Female | Male |  |  |  |  |  | Female | Male |  | |  | |  |
|  | 2.48 | 2.01 | | 7.20 | 7.01 | | 2.03 | | 1.89 | | 8.05 | | 7.01 | |
|  |  | 2.02 | | 7.20 | 7.16 | |  | |  | | 8.06 | | 6.92 | |
|  |  |  | | 8.01 | 7.15 | |  | |  | | 8.06 | | 7.10 | |
|  |  | 1.31 | | 7.11 | 6.12 | | 2.01 | | 1.03 | | 5.01 | | 6.08 | |
|  |  | 1.42 | | 6.90 | 6.03 | |  | |  | | 5.00 | | 6.01 | |
|  |  | 1.74 | | 7.00 | 7.28 | |  | |  | | 5.03 | | 5.92 | |
|  | 2.45 | 1.03 | | 4.60 | 5.01 | | 1.41 | | 0.98 | | 4.01 | | 5.71 | |
|  |  |  | | 4.41 | 5.00 | |  | | 1.01 | | 5.02 | | 5.32 | |
|  | 2.32 | 1.50 | | 4.71 | 7.17 | | 1.97 | | 1.89 | | 4.96 | | 5.81 | |
|  |  | 1.61 | | 5.28 | 6.30 | |  | |  | | 5.01 | | 6.17 | |
|  | 2.37 | 1.42 | | 8.01 | 8.31 | | 1.01 | | 1.01 | | 7.12 | | 8.01 | |
|  |  |  | |  | 7.01 | | 0.96 | |  | |  | | 7.96 | |
|  | 2.11 |  | | 7.91 | 7.45 | | 0.98 | | 0.91 | | 8.35 | | 7.04 | |
|  | 2.27 |  | | 8.07 | 7.47 | |  | |  | | 8.44 | | 6.83 | |
|  | 2.40 | 2.11 | | 6.21 | 6.42 | |  | | 0.89 | | 5.72 | | 6.21 | |
|  | 2.42 |  | | 6.10 |  | |  | |  | | 5.34 | |  | |
|  | 2.43 | 1.98 | | 6.02 | 6.18 | | 0.98 | | 0.87 | | 5.44 | | 5.00 | |
|  |  |  | | 6.01 |  | |  | | 1.10 | | 5.54 | | 6.01 | |
|  | 2.33 | 2.98 | | 5.21 | 5.12 | | 1.49 | | 1.01 | | 5.32 | | 6.11 | |
|  | 2.29 |  | | 5.19 | 4.72 | |  | |  | |  | |  | |
|  | 2.54 | 2.49 | | 6.42 | 6.48 | | 1.51 | | 1.40 | | 6.99 | | 7.82 | |
|  |  |  |  |  |  |  |  |  |  |  |  |  | 8.07 | |
|  | 3.43 | 1.42 | | 7.98 | 8.23 | | 1.31 | | 1.29 | | 6.98 | | 7.02 | |
|  |  | 1.39 | |  |  |  |  |  |  |  |  |  |  |  |
|  |  | 1.40 | |  |  |  |  |  |  |  |  |  |  |  |
|  | 4.98 | 1.91 | | 8.99 | 8.42 | |  | | 1.97 | | 4.93 | | 5.17 | |
|  |  | 2.07 | |  |  |  |  |  |  |  | 4.87 | | 5.20 | |
|  |  |  |  |  |  |  |  |  |  |  | 5.16 | | 5.58 | |
|  | 2.00 | 1.70 | | 4.04 | 5.03 | | 0.99 | | 1.92 | | 4.71 | | 3.11 | |
|  | 1.90 | 1.81 | | 5.04 | 4.98 | |  |  |  |  | 4.81 | |  |  |
|  |  |  |  | 5.04 | 7.00 | |  |  |  |  | 5.35 | |  |  |
|  | 3.12 | 1.55 | | 4.32 | 2.20 | | 1.98 | | 1.97 | | 7.96 | | 5.82  5.20 | |
|  |  | 1.53 | | 5.25 | 3.22 | |  |  |  |  |  |  |  |  |
|  |  | 2.05 | | 5.42 |  | |  |  |  |  |  |  |  |  |
|  | 2.32 | 1.50 | | 7.90 | 8.03 | | 2.01 | | 0.98 | | 7.01 | | 5.12 | |
|  | 2.10 | 1.48 | |  |  |  |  |  |  |  |  |  |  |  |
|  |  | 2.05 | |  |  |  |  |  |  |  |  |  |  |  |
|  | 9.46 | 1.47 | | 6.98 | 6.42 | |  | | 1.57 | | 7.02 | | 6.38 | |
|  |  |  |  |  |  |  |  |  | 1.40 | |  |  | 6.60 | |
|  | 1.71 | 2.00 | | 7.89 | 8.94 | | 1.96 | | 0.97 | | 6.99 | | 7.01 | |
|  | 1.78 | 2.02 | |  |  |  |  |  |  |  |  |  | 6.00 | |
|  | 2.12 | 1.50 | | 4.21 | 5.18 | | 2.03 | | 1.98 | | 4.82 | | 5.12 | |
|  | 2.30 | 1.59 | | 5.17 | 5.31 | |  |  |  |  | 4.90 | | 5.08 | |
|  |  | 1.42 | | 5.59 | 5.40 | |  |  |  |  | 5.25 | | 6.73 | |
|  | 2.18 | 1.52 | | 5.72 | 5.38 | | 2.00 | | 1.97 | | 5.98 | | 5.95 | |
|  | 2.20 | 1.51 | | 6.17 | 5.66 | |  |  |  |  |  |  | 6.01 | |
|  | 1.70 | 2.00 | | 3.42 | 4.06 | | 1.98 | | 0.92 | | 3.41 | | 5.27 | |
|  | 1.76 | 1.99 | |  |  |  |  |  |  |  |  |  |  |  |
|  | 3.17 | 2.01 | | 3.91 | 5.27 | | 1.67 | | 0.97 | | 7.01 | | 6.45  6.44 | |
|  |  |  |  | 5.01 | 6.12 | |  |  |  |  |  |  |  |  |
|  |  |  |  | 5.11 | 5.50 | |  |  |  |  |  |  |  |  |
|  | 2.20 | 1.93 | | 5.89 | 7.21 | | 0.92 | | 0.88 | | 7.97 | | 7.86 | |
|  | 2.11 | 1.95 | | 6.12 |  |  | 0.95 | |  |  |  |  |  |  |
|  | 3.31 | 2.98 | | 8.03 | 8.67 | | 1.31 | | 0.73 | | 7.13 | | 7.32 | |
|  |  |  |  |  |  |  |  |  | 0.83 | |  |  |  |  |
|  | 2.71 | 2.12 | | 5.30 | 6.31 | | 0.99 | | 0.87 | | 6.97 | | 8.04 | |
|  | 2.61 | 2.20 | | 5.00 | 4.56 | |  |  |  |  |  |  |  |  |
|  | 2.89 | 2.03 | | 8.02 | 7.99 | | 1.31 | | 1.98 | | 5.86 | | 7.22 | |
|  |  |  |  |  |  |  |  |  |  |  | 5.10 | |  |  |
|  | 2.80 | 1.51 | | 7.01 | 8.30 | | 2.31 | | 2.03 | | 5.11 | | 5.51 | |
|  | 2.71 | 1.62 | | 6.40 |  |  |  |  |  |  | 4.89 | | 5.40 | |
|  |  | 1.76 | |  |  |  |  |  |  |  | 4.37 | |  |  |
|  | 2.40 | 1.91 | | 5.71 | 5.03 | | 2.51 | | 2.20 | | 5.01 | | 5.13 | |
|  |  | 2.07 | | 7.00 | 6.04 | |  | | 2.21 | | 4.72 | | 4.89 | |
|  |  |  |  |  |  |  |  |  |  |  | 4.58 | | 4.45 | |
|  | 2.89 | 1.88 | | 5.91 | 8.71 | | 2.42 | | 2.51 | | 3.87 | | 6.12 | |
|  |  |  |  | 6.00 |  |  |  |  |  |  | 4.02 | | 5.59 | |
|  | 2.91 | 2.03 | | 6.70 | 7.43 | | 2.67 | | 2.38 | | 5.37 | | 8.16 | |
|  | 2.96 |  |  | 6.61 | 7.90 | |  |  |  |  | 6.50 | | 7.14 | |
|  | 3.14 | 3.03 | | 7.88 | 4.98 | | 2.89 | | 2.68 | | 5.21 | | 5.42 | |
|  |  |  |  |  | 5.03 | |  |  |  |  | 5.15 | | 5.25 | |
| Σ | 112.28 | 93.53 | | 338.31 | 321.90 | | 48.56 | | 51.20 | | 305.46 | | 339.87 | |
| n | 42 | 51 | | 55 | 51 | | 29 | | 35 | | 53 | | 55 | |
| $\bar{x}$ | 2.67 | 1.83 | | 6.15 | 6.31 | | 1.67 | | 1.46 | | 5.76 | | 6.18 | |
| SD | 1.22 | 0.41 | | 1.31 | 1.47 | | 0.58 | | 0.58 | | 1.29 | | 1.05 | |
