# Supplementary material for: Adding Perches for Cross-Pollination Ensures the Reproduction of a Self-Incompatible Orchid
Source: PLoS One. 2013 Jan 7;8(1):e53695. doi: 10.1371/journal.pone.0053695 (PMC3538729; doi:10.1371/journal.pone.0053695)
Supplement: Table S4 — Rate of natural fruit setting of inflorescence with pollen removed and pollen present in C. rigida . (DOC) [file pone.0053695.s008.doc]

***Table S4****. Rate of natural fruit setting of inflorescence with pollen removed and pollen present in C. rigida*

| Sample pair | Pollen removed | | | | Pollen present | | |
| --- | --- | --- | --- | --- | --- | --- | --- |
|  | No. of flowers | No. of capsules | Rate of fruit set |  | No. of flowers | No. of capsules | Rate of fruit set |
|  | 28 | 8 | 28.57 | | 28 | 6 | 21.43 |
|  | 22 | 12 | 54.55 | | 26 | 6 | 23.08 |
|  | 22 | 11 | 50.00 | | 30 | 4 | 13.33 |
|  | 24 | 10 | 41.67 | | 30 | 10 | 33.33 |
|  | 30 | 12 | 40.00 | | 26 | 4 | 15.38 |
|  | 22 | 8 | 36.36 | | 28 | 7 | 25.00 |
|  | 24 | 9 | 37.50 | | 24 | 6 | 25.00 |
|  | 22 | 5 | 22.73 | | 30 | 8 | 26.67 |
|  | 20 | 6 | 30.00 | | 26 | 7 | 26.92 |
|  | 22 | 14 | 63.64 | | 26 | 6 | 23.08 |
|  | 27 | 7 | 25.93 | | 28 | 9 | 32.14 |
|  | 26 | 6 | 23.08 | | 27 | 6 | 22.22 |
|  | 28 | 6 | 21.43 | | 30 | 4 | 13.33 |
|  | 31 | 7 | 22.58 | | 28 | 4 | 14.29 |
|  | 20 | 3 | 15.00 | | 26 | 5 | 19.23 |
|  | 22 | 5 | 22.73 | | 27 | 6 | 22.22 |
|  | 21 | 6 | 28.57 | | 25 | 5 | 20.00 |
|  | 24 | 6 | 25.00 | | 24 | 3 | 12.50 |
|  | 23 | 5 | 21.74 | | 25 | 4 | 16.00 |
|  | 23 | 6 | 26.09 | | 29 | 5 | 17.24 |
|  | 20 | 7 | 35.00 | | 27 | 5 | 18.52 |
|  | 23 | 7 | 30.43 | | 25 | 4 | 16.00 |
|  | 24 | 5 | 20.83 | | 31 | 6 | 19.35 |
|  | 30 | 6 | 20.00 | | 27 | 5 | 18.52 |
|  | 28 | 5 | 17.86 | | 23 | 3 | 13.04 |
|  | 20 | 5 | 25.00 | | 25 | 3 | 12.00 |
|  | 27 | 7 | 25.93 | | 28 | 5 | 17.86 |
|  | 21 | 4 | 19.05 | | 30 | 6 | 20.00 |
|  | 29 | 8 | 27.59 | | 31 | 4 | 12.90 |
|  | 21 | 12 | 57.14 | | 24 | 2 | 8.33 |
| $\bar{x}$ | 24.13 | 7.27 | 30.53 | | 27.13 | 5.27 | 19.30 |
| SD | 3.39 | 2.69 | 12.26 | | 2.27 | 1.78 | 5.96 |
