# Supplementary material for: Adding Perches for Cross-Pollination Ensures the Reproduction of a Self-Incompatible Orchid
Source: PLoS One. 2013 Jan 7;8(1):e53695. doi: 10.1371/journal.pone.0053695 (PMC3538729; doi:10.1371/journal.pone.0053695)
Supplement: Table S5 — Observation results of pollination experiments on the mating system of C. fimbriatum . (DOC) [file pone.0053695.s009.doc]

***Table S5.*** *Observation results of pollination experiments on the mating system of C. fimbriatum*

| Sample  pair | Natural pollination | | | | Artificial self-pollination | | | | Artificial cross-pollination | | |
| --- | --- | --- | --- | --- | --- | --- | --- | --- | --- | --- | --- |
|  | No. of flowers | No. of capsules | Rate of fruit set |  | No. of flowers | No. of capsules | Rate of fruit set |  | No. of flowers | No. of capsules | Rate of fruit set |
|  | 20 | 0 | 0 | | 20 | 0 | 0 | | 20 | 15 | 75 |
|  | 20 | 1 | 5 | | 20 | 0 | 0 | | 20 | 16 | 80 |
|  | 20 | 0 | 0 | | 20 | 0 | 0 | | 20 | 17 | 85 |
|  | 20 | 0 | 0 | | 20 | 0 | 0 | | 20 | 13 | 65 |
|  | 20 | 0 | 0 | | 20 | 0 | 0 | | 20 | 14 | 70 |
|  | 20 | 0 | 0 | | 20 | 0 | 0 | | 20 | 18 | 90 |
|  | 20 | 0 | 0 | | 20 | 0 | 0 | | 20 | 15 | 75 |
|  | 20 | 0 | 0 | | 20 | 0 | 0 | | 20 | 12 | 60 |
|  | 20 | 0 | 0 | | 20 | 0 | 0 | | 20 | 14 | 70 |
|  | 20 | 0 | 0 | | 20 | 0 | 0 | | 20 | 15 | 75 |
|  | 20 | 0 | 0 | | 20 | 0 | 0 | | 20 | 16 | 80 |
|  | 20 | 0 | 0 | | 20 | 0 | 0 | | 20 | 17 | 85 |
|  | 20 | 0 | 0 | | 20 | 0 | 0 | | 20 | 11 | 55 |
|  | 20 | 1 | 5 | | 20 | 0 | 0 | | 20 | 10 | 50 |
|  | 20 | 0 | 0 | | 20 | 0 | 0 | | 20 | 13 | 65 |
|  | 20 | 0 | 0 | | 20 | 0 | 0 | | 20 | 17 | 85 |
|  | 20 | 0 | 0 | | 20 | 0 | 0 | | 20 | 15 | 75 |
|  | 20 | 0 | 0 | | 20 | 0 | 0 | | 20 | 16 | 80 |
|  | 20 | 0 | 0 | | 20 | 0 | 0 | | 20 | 14 | 70 |
|  | 20 | 0 | 0 | | 20 | 0 | 0 | | 20 | 13 | 65 |
| $\bar{x}$ | 20 | 0.1 | 0.5 | | 20 | 0 | 0 | | 20 | 14.55 | 72.75 |
| SD | 0 | 0.31 | 1.54 | | 0 | 0 | 0 | | 0 | 2.11 | 10.57 |
